# The GTPase RAB6 is required for stem cell maintenance and cell migration in the gut epithelium

**DOI:** 10.1242/dev.203038

**Published:** 2024-10-21

**Authors:** Pierre Simonin, Gehenna Lobo Guerrero, Sabine Bardin, Ram Venkata Gannavarapu, Denis Krndija, Joseph Boyd, Stephanie Miserey, Danijela Matic Vignjevic, Bruno Goud

**Affiliations:** Institut Curie, PSL Research University, CNRS UMR 144, F-75005 Paris, France

**Keywords:** RAB GTPases, Gut epithelium, Migration, Stem cells, Mouse models

## Abstract

Intestinal epithelial cells, which are instrumental in nutrient absorption, fluid regulation, and pathogen defense, undergo continuous proliferation and differentiation within the intestinal crypts, migrating towards the luminal surface where they are eventually shed. RAB GTPases are key regulators of intracellular vesicular trafficking and are involved in various cellular processes, including cell migration and polarity. Here, we investigated the role of RAB6 in the development and maintenance of the gut epithelium. We generated conditional knockout mice with RAB6 specifically deleted in the gut epithelium. We found that deletion of the *Rab6a* gene resulted in embryonic lethality. In adult mice, RAB6 depletion led to altered villus architecture and impaired junction integrity without affecting the segregation of apical and basolateral membrane domains. Further, RAB6 depletion slowed down cell migration and adversely affected both cell proliferation and stem cell maintenance. Notably, the absence of RAB6 resulted in a diminished number of functional stem cells, as evidenced by the rapid death of isolated crypts from *Rab6a* KO mice when cultured as 3D organoids. Together, these results underscore the essential role of RAB6 in maintaining gut epithelial homeostasis.

## Teaser

**Summary:** Deletion of RAB6, a key regulator of intracellular transport in the mouse gut epithelium reveals that this protein is required for the maintenance of gut epithelial homeostasis.

## INTRODUCTION

Intestinal epithelial cells form a single layer of epithelium, which plays crucial roles in nutrient absorption, fluid balance and protection against pathogens. They continuously proliferate at the base of intestinal crypts. After certain rounds of division, on their way out of the crypt, they differentiate into various cell types, including enterocytes, goblet cells and Paneth cells. Following differentiation, epithelial cells migrate along the crypt-villus axis towards the luminal surface of the intestine (Krndija et al., 2019). They migrate collectively, with cells remaining tightly connected to one another to prevent the leakage of luminal contents into the underlying tissue. Upon reaching the luminal surface, these cells ultimately undergo apoptosis and are shed into the intestinal lumen. This process of cell extrusion is counterbalanced by the continuous proliferation and differentiation of stem cells and their subsequent migration.

RAB GTPases form a large family of proteins (over 60 in humans) that are key regulators of intracellular vesicular traffic (Hutagalung and Novick, 2011). The RAB6 sub-family consists of four proteins named RAB6A, RAB6A′, RAB6B and RAB6C. RAB6A′ is generated by alternative splicing of the *RAB6A* gene and differs from RAB6A by only three amino acids (Echard et al., 2000). Both proteins are ubiquitously expressed and localized to membranes of the Golgi apparatus and the *trans*-Golgi network (TGN) (Goud et al., 2018). RAB6B is encoded by a separate gene and is mostly expressed in neurons and neuroendocrine cells (Opdam et al., 2000). RAB6C is a primate-specific retrogene transcribed in a limited number of human tissues. It localizes to the centrosome and is involved in cell cycle progression (Young et al., 2010).

RAB6 regulates several transport steps at the level of the Golgi complex, including transport of all types of cargo between the Golgi complex and the plasma membrane (Fourriere et al., 2019) and a retrograde route that connects the plasma membrane and the Golgi complex via endosomes (Goud et al., 2018). In relation to its functions in transport, RAB6 has been shown to play a key role in other cellular processes. For instance, the retrograde route is used to recycle beta-1 integrins from the plasma membrane to the leading edge of migratory cells, a process important for persistent cell migration (Shafaq-Zadah et al., 2016). In neural stem cells (radial glial cells), which are highly polarized, RAB6 and dynein drive post-Golgi apical transport of polarity components such as Crumbs. Impairing this pathway disrupts neuroepithelial integrity (Brault et al., 2022). The integrity and homeostasis of the gut epithelium thus rely on several processes that could require RAB6 function, making it a good model in which to investigate the physiological role of RAB6.

The invalidation of the *Rab6a* gene, which leads to the depletion of both RAB6A and RAB6A′ in all tissues, is embryonic lethal (Bardin et al., 2015; Shafaq-Zadah et al., 2016). Here, we generated conditional knockout (KO) mice in which *Rab6a* is specifically deleted in the gut epithelium. We found that the absence of RAB6A/A′ specifically from the intestinal epithelium also leads to embryonic lethality. Moreover, in adult mice, RAB6 deletion results in disruption of the villus architecture, impairs the epithelial cell migration and adversely affects both cell proliferation and the maintenance of stem cells.

## RESULTS

### Depletion of RAB6 from intestinal epithelium is embryonically lethal

To investigate the role of RAB6 in the gut epithelium, we generated a conditional KO of *Rab6a* by crossing *Rab6a* floxed mice with Villin-Cre transgenic mice. The villin (*Vil1*) promoter, active from 9 days post-coitum (el Marjou et al., 2004), drives Cre recombinase expression in most intestinal cell types, including stem cells, inducing *Rab6a* deletion in gut epithelium.

Of the 60 offspring from these mouse intercrosses, 25% were expected to be *Rab6a*^*−*/*−*^ but none of them survived to birth, indicating that the absence of *Rab6a* in the gut epithelium results in embryonic lethality (Table S1). There is evidence that RAB6B, the neuronal isoform of RAB6, can compensate for the function of RAB6A/A′ (Homma et al., 2019; Brault et al., 2022). However, RAB6B is either not expressed or present at a very low level in intestinal epithelial cells (Iwaki et al., 2020). In addition, the phenotype of organoids derived from double KO mice (*Rab6a*+*Rab6b*) (Brault et al., 2022) was similar to that obtained with *Rab6a* KO mice (see below).

**Fig. 1.**
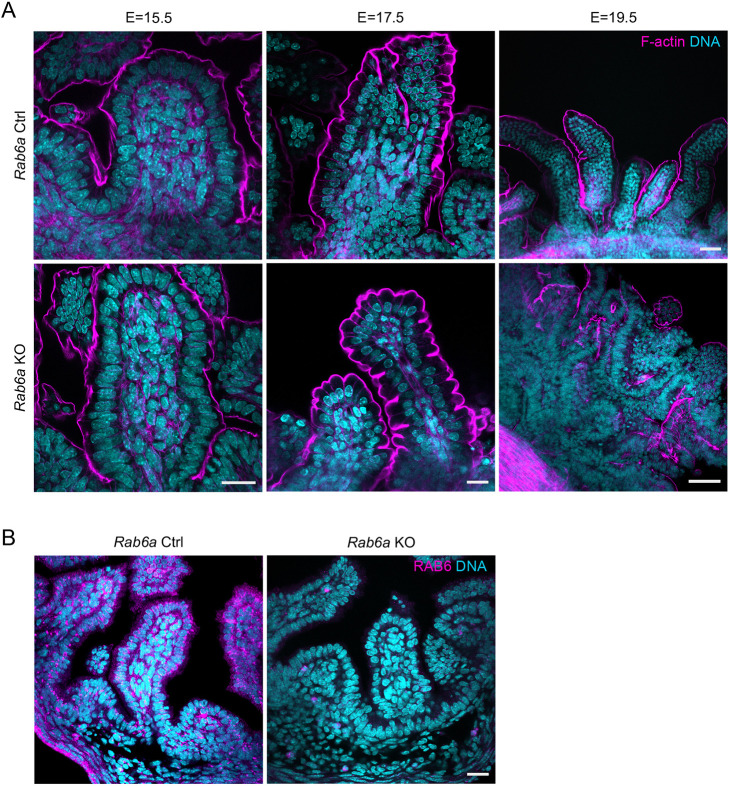
**RAB6 depletion in the gut epithelium leads to the disorganization of intestinal epithelium in embryos.** (A) F-actin (phalloidin) staining at E15.5, E17.5 and E19.5 in *Rab6a* control (Ctrl) and *Rab6a* KO embryos. The intestinal epithelium is disorganized at E19.5 in *Rab6a* KO mice. Nuclei were visualized with DAPI staining. Scale bars: 20 µm (E15.5 and E17.5); 50 µm (E19.5). (B) Immunostaining of RAB6 at E17.5 in *Rab6a* Ctrl and *Rab6a* KO mice. Only a few cells remained RAB6 positive. Scale bar: 20 µm.

Morphological analysis of the gut in embryonic stages revealed a normal appearance up embryonic day (E) 17.5 (Fig. 1A). At this stage, RAB6 (herein referring to both RAB6A/A′ and RAB6B) was entirely depleted in most epithelial cells as monitored by immunofluorescence (Fig. 1B). A few remaining RAB6-positive cells likely correspond to cells that do not express villin, such as tuft cells (Esmaeilniakooshkghazi et al., 2020), or enteroendocrine cells expressing RAB6B, and thus were not affected by the RAB6 depletion strategy. At E19.5, the intestinal epithelium appeared disorganized, which likely explains the embryonic lethality (Fig. 1A, Table S1).

Together, these experiments show that RAB6 has an essential role in the development and organization of the gut epithelium, with its absence leading to embryonic lethality, likely due to disruption of the intestinal epithelial structure.

### Villus architecture is altered in adult *Rab6a* KO mice

**Fig. 2.**
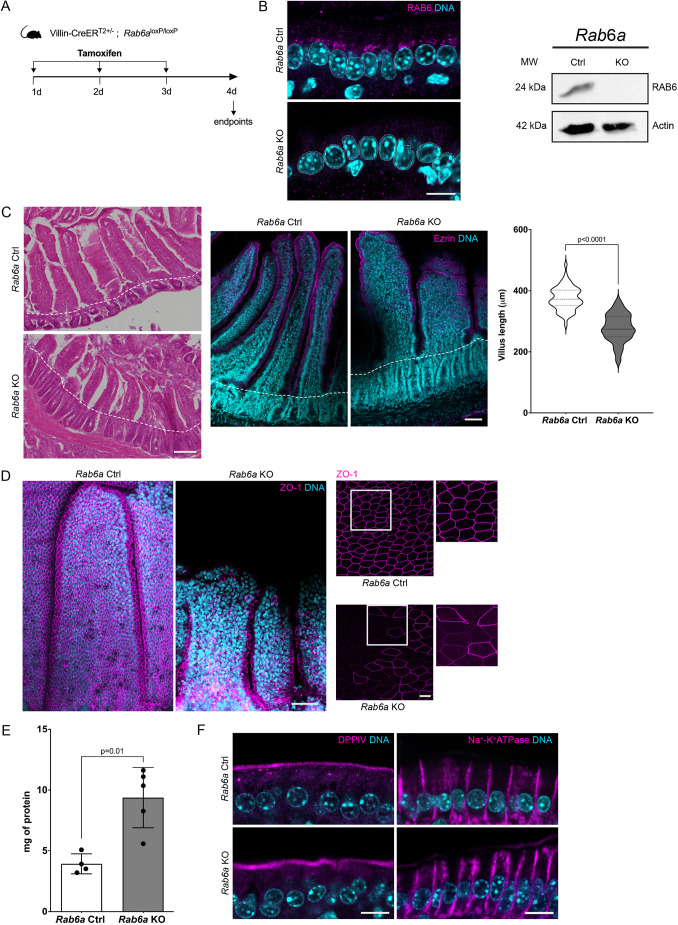
**Villus architecture is altered following RAB6 depletion.** (A) Experimental scheme for RAB6 depletion. Mice were injected intraperitoneally with tamoxifen once a day for three consecutive days and sacrificed at day 4. (B) Validation of RAB6 depletion by immunostaining and western blotting; actin was used as a loading control. MW, molecular weight. Scale bar: 10 µm; (C) Left: Hematoxylin/Eosin staining of small intestine sections from *Rab6a* control (Ctrl) and *Rab6a* KO mice. Scale bar: 100 μm. Middle: Small intestine of control and *Rab6a* KO mice, stained for ezrin (magenta) and DNA (DAPI; cyan). Scale bar: 50 μm. Right: Quantification of villus length (*n*=60 villi). Data are presented as violin plots and analyzed by two-tailed unpaired Student's *t*-test. Dashed lines mark the boundary between crypt and villus. (D) Immunostaining of the tight junction marker ZO-1. Images are sum projections. Higher magnifications (boxes) are displayed on the right. Scale bars: 50 μm (main panels); 10 μm (insets). (E) In order to assess cell adhesion strength, protein concentration of epithelial cell lysates from small intestines tissues was quantified using the detachment assay (see Materials and Methods for details). Data were analyzed by Mann–Whitney test; error bars indicate s.d. (F) Immunostaining of DPPIV (apical) and Na^+^-K^+^ ATPase (lateral) polarity markers. Their localization was not affected by RAB6 depletion. Scale bars: 10 µm.

We then sought to examine the role of RAB6 in the mature gut epithelium during gut homeostasis. We generated inducible *Rab6a* KO mice by crossing *Rab6a* floxed mice with Villin-CreER^T2^ mice. RAB deletion was induced in adult, 3-month-old mice, injected with tamoxifen for three consecutive days (Fig. 2A,B).

Following RAB6 depletion, the overall crypt-villus architecture of the gut epithelium was globally maintained, although villi were significantly shorter compared with those in control mice (Fig. 2C). The epithelial apical surface was also more undulated (Fig. 2B). Alterations of the localization of the tight junction protein ZO-1 (TJP1) were significant, including complete absence in some regions of the epithelium and a marked reduction in overall intensity of the ZO-1 signal (Fig. 2D). These observations suggest that the integrity of tight junctions was compromised, aligning with the presence of fractures within the epithelium. In addition, *Rab6a* KO cells were more loosely attached to the basement membrane, as demonstrated by a cell detachment assay (De Arcangelis et al., 2017), indicating a decrease in cell adhesion properties (Fig. 2E). Despite these structural alterations, the localization of basement membrane proteins (collagen type IV and laminin α5) was not affected (Fig. S1A). In addition, the number of apoptotic cells (stained with cleaved caspase 3) at the tips of the villi, remained unchanged between *Rab6a* KO and control intestinal epithelial cells (Fig. S1B).

We next investigated the localization of apical and basolateral proteins (Fig. 2F). The staining of DPPIV (DPP4), a GPI-anchored protein localized at the apical membrane, and the localization of Na^+^-K^+^ ATPase at the basolateral membrane showed no significant difference from control, suggesting that the depletion of RAB6 does not alter apical and basolateral transport pathways. Together, this analysis underscores the role of RAB6 in maintaining the structural and functional integrity of the gut epithelium in adult mice, highlighting specific alterations in villus morphology, tight junction integrity, and epithelial cohesion without markedly affecting the segregation of apical and basolateral membrane domains.

### RAB6 depletion slows down epithelial cell migration

**Fig. 3.**
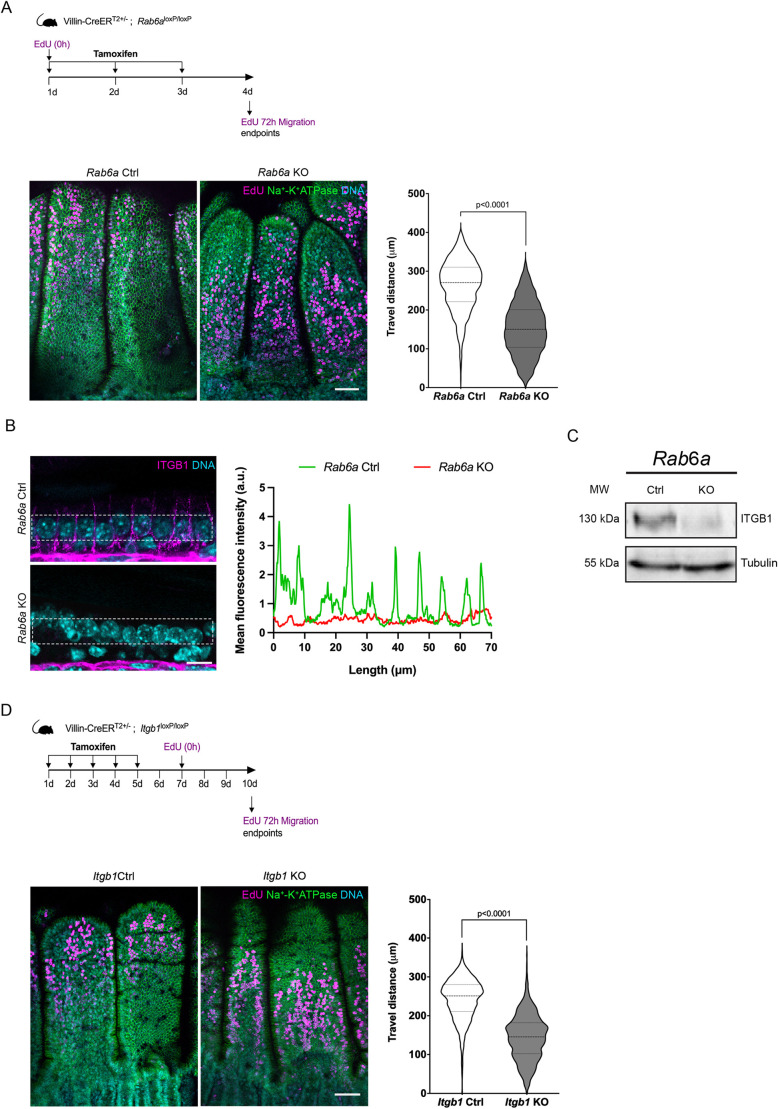
**Loss of RAB6 and β1 integrin impairs intestinal epithelial cell migration.** (A) Top: Experimental scheme of the migration assay in *Rab6a* KO mice. EdU was injected 72 h before sacrifice. Bottom: EdU labeling and quantification of the travel distance to the tip of the villi of EdU-positive cells in *Rab6a* control (Ctrl) and *Rab6a* KO mice (*n*=30 villi). Scale bar: 50 µm. (B) Immunostaining of ITGB1. The lateral pool of β1 integrin was quantified using a line scan across the region of interest (dashed areas). The ITGB1 mean fluorescence intensity is almost undetectable in the *Rab6a* KO section. Scale bar: 10 µm. a.u., arbitrary units. (C) Immunoblotting to assess the total level of ITGB1 in *Rab6a* Ctrl and *Rab6a* KO mice; tubulin was used as a loading control. MW, molecular weight. (D) Top: Experimental scheme of the migration assay in *Itgb1* KO mice. EdU was injected 72 h before sacrifice. Bottom: EdU labeling and quantification of the travel distance to the tip of the villi in *Itgb1* Ctrl and *Itgb1* KO mice (*n*=30 villi). Scale bar: 50 µm. Data are presented as violin plots and analyzed by two-tailed unpaired Student's *t*-test.

Epithelial cells migrate collectively from the bottom to the top of the villus using actin-based protrusions (Krndija et al., 2019). To investigate whether RAB6 depletion affects cell migration, we performed 5-ethynyl-2′-deoxyridine (EdU) pulsed-chase assays over 72 h. We found that cells were significantly slower in *Rab6a* KO mice (Fig. 3A).

We previously showed that RAB6 and a retrograde transport pathway connecting the plasma membrane with the Golgi complex play a crucial role in recycling a pool of β1 integrins (ITGB1). Recycling of integrins is pivotal for persistent cell migration (Shafaq-Zadah et al., 2016). In intestinal epithelial cells, β1 integrins are predominantly localized at the basolateral membrane (Lussier et al., 2000) (Fig. 3B). Remarkably, this localization was lost in *Rab6a* KO mice (Fig. 3B), suggesting that the migratory defect observed in *Rab6a* KO mice may originate from impaired β1 trafficking. In addition, the total level of ITGB1, assessed by immunoblotting, was reduced in *Rab6a* KO mice (Fig. 3C).

To assess directly the contribution of β1 integrin in epithelial cell migration, we generated intestinal epithelium-specific *Itgb1* KO mice by crossing *Itgb1* floxed mice with Villin-Cre^ERT2^ mice. Treatment with tamoxifen for five consecutive days, followed by a 5-day resting period, resulted in a strong depletion of β1 integrin in epithelial cells observed by immunofluorescence and western blotting (Fig. S2A,B). The intestinal epithelium phenotype after β1 integrin depletion showed a strong resemblance to that observed in *Rab6a* KO mice, including the overall preservation of the crypt-villus architecture (Fig. 3D), easier detachment from the basement membrane (Fig. S2C), and undulation of the apical surface (Fig. S2D). The localization of apical (DPPIV) and basolateral (Na^+^-K^+^ ATPase) markers was also unaffected (Fig. S2E). However, the alteration of ZO-1 localization was less pronounced than that observed in *Rab6a* KO mice (Fig. S2F).

EdU pulsed-chase experiments in *Itgb1* KO mice showed that the depletion of β1 integrin impaired the migration of cells to a similar extent as RAB6 depletion (Fig. 3D). During migration, cell surface-associated integrins undergo constant cycles of endocytosis and recycling (Moreno-Layseca et al., 2019). To test directly whether RAB6 participates in integrin recycling, we performed endocytosis/recycling assays using organoids derived from *Rab6a* KO mice. β1 integrin showed a similar distribution pattern in organoids and *in vivo* epithelium (Fig. S3A). The treatment of organoids *in vitro* with 4-hydroxytamoxifen (4-OHT) resulted in robust RAB6 depletion (Fig. S3B). Following incubation on ice with pan anti-β1 antibodies (CD29), we monitored recycling after internalization of bound antibodies for 30 min at 37°C in organoids depleted, or not, for RAB6. Surprisingly, there was no significant difference in the amount of integrin recycled back to the plasma membrane after internalization between *Rab6a* KO and control organoids (Fig. S4). Previous studies have implicated RAB6 and the retrograde pathway in the recycling of inactive β1 integrin (Shafaq-Zadah et al., 2016). Although a specific antibody for detecting inactive mouse integrin is unavailable, the existing evidence indicates that most of the integrin at the cell surface is in its inactive conformation (Arjonen et al., 2012). Consequently, this finding suggests that RAB6 may not play a significant role in recycling β1 integrin in intestinal epithelial cells.

### RAB6 depletion impacts both proliferation and stem cell maintenance

**Fig. 4.**
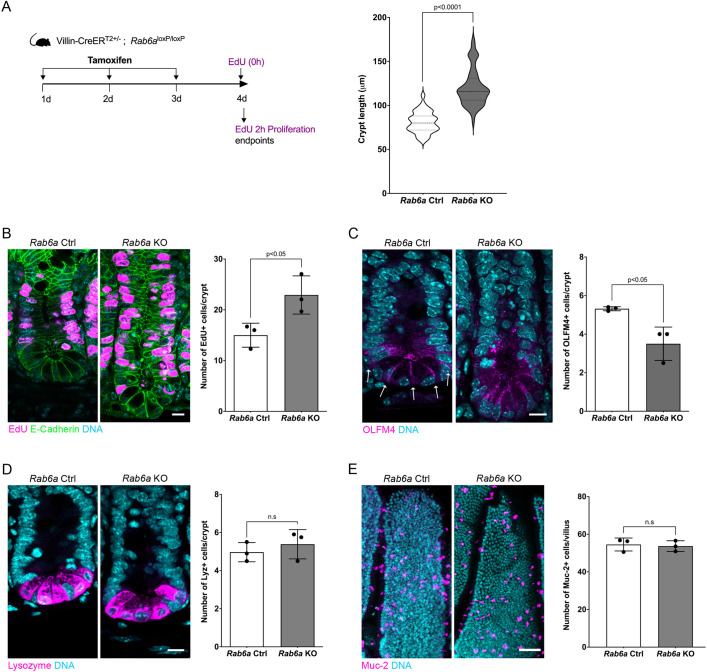
**RAB6 depletion impacts both proliferation and stem cell maintenance.** (A) Left: Experimental scheme of the proliferation assay in *Rab6a* KO mice. EdU was injected 2 h before sacrifice. Right: Crypt length quantification (*n*=75 crypts). Data are presented as violin plots and analyzed by two-tailed unpaired Student's *t*-test. (B) EdU labeling and quantification of the number of EdU-positive cells in *Rab6a* control (Ctrl) and *Rab6a* KO (*n*=60 crypts). Scale bar: 10 µm. (C) Immunostaining and quantification of stem cells (OLFM4^+^) from *Rab6a* Ctrl and *Rab6a* KO (*n*=60 crypts). Arrows indicate the expected OLFM4 staining. Scale bar: 10 µm; (D) Immunostaining and quantification of Paneth cells (lysozyme^+^) in *Rab6a* Ctrl and *Rab6a* KO (*n*=60 crypts). Scale bar: 10 µm. (E) Immunostaining and quantification of goblet cells (mucin 2, Muc2^+^) in *Rab6a* Ctrl and *Rab6a* KO (*n*=30 villi). Scale bar: 50 µm. In B-E, data were analyzed by two-tailed unpaired Student's *t*-test; error bars indicate s.d. n.s., not significant.

To examine the impact of RAB6 depletion on proliferation and stem cell maintenance, we next focused on the crypt. Histological analysis revealed that crypts in *Rab6a* KO mice were longer compared with those in control mice (Fig. 4A). This morphological alteration correlated with a significant increase in EdU incorporation, indicating heightened cell proliferation in *Rab6a* KO mice (Fig. 4B). We also observed a higher number of phospho-histone H3-positive cells, reflecting higher number of mitotic cells (Fig. S5).

To determine whether RAB6 depletion affects stem cells, we stained crypts with the stem cell marker OLFM4. Strikingly, OLFM4 staining was altered in *Rab6a* KO mice, and the number of OLFM4-positive cells was decreased (Fig. 4C). In addition, we observed a higher number of apoptotic cells, labeled with cleaved caspase 3 in crypts of *Rab6a* KO mice (Fig. S1C). These results indicate the necessity of RAB6 for the maintenance of stem cells (Fig. 4C). However, the continued proliferation of crypt cells suggests that transit-amplifying cells – those derived from stem cells and characterized by rapid proliferation and differentiation – were not affected by RAB6 depletion.

To determine whether differentiation of transit-amplifying cells is affected by RAB6 depletion, we stained tissue with an anti-Muc2 antibody to label goblet cells and a lysozyme antibody to label Paneth cells. We found that the number of goblet and Paneth cells was comparable in *Rab6a* KO and control mice (Fig. 4D,E).

**Fig. 5.**
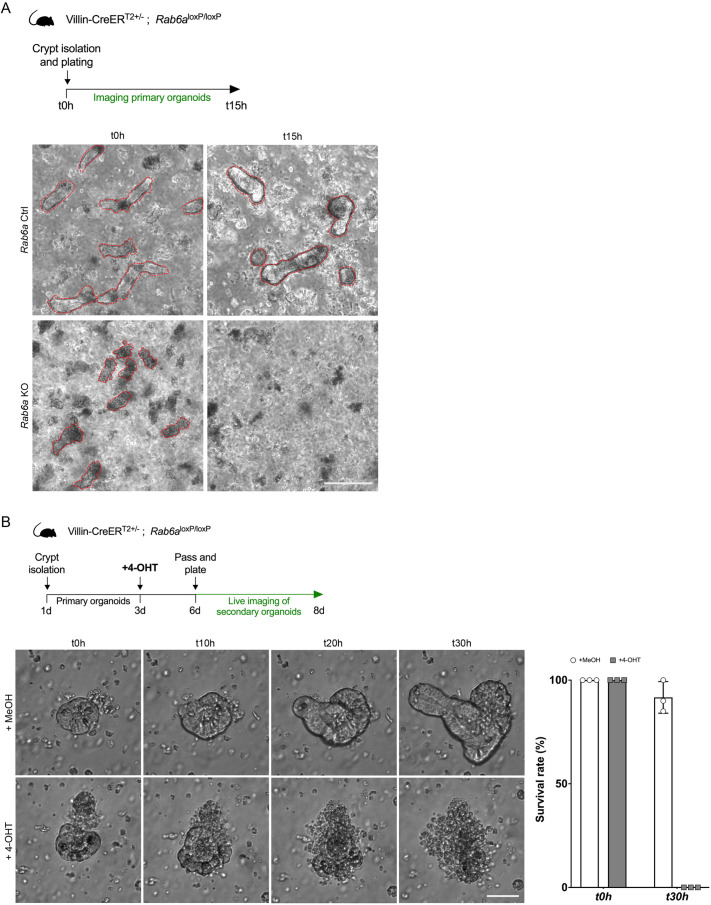
**RAB6 function is crucial for the development of primary or secondary gut organoids.** (A) Crypts were isolated from *Rab6a* control (Ctrl) and *Rab6a* KO mice and plated. The development of primary organoids was followed by imaging. Images show *Rab6a* KO crypts dying at t=0 h and at t=15 h no crypts are observed. In comparison, Ctrl crypts develop normally. Red dashed lines outline crypts. Scale bar: 100 µm. (B) Crypts were isolated from *Rab6a*^+/−^ mice and plated. To induce RAB6 depletion *in vitro*, organoids were treated at day 3 with 2 µm 4-OHT or with methanol (MeOH) for control. At day 6, organoids were split and plated again to follow the development of secondary organoids by live imaging. Images show that Ctrl crypts (MeOH) follow a normal growth, whereas *Rab6a* KO crypts (4-OHT) are dying 20 h after splitting. Graph shows the survival rate of crypts from t=0 h to t=30 h (*n*=30 crypts). Scale bar: 50 µm.

To analyze whether stem cells in *Rab6a* KO mice are functional, we isolated crypts from control and *Rab6a* KO mice. Whereas crypts from control mice developed into 3D organoids, those from *Rab6a* KO mice rapidly died after plating (Fig. 5A), suggesting that the absence of RAB6 results in a reduced number of functional stem cells. Furthermore, to assess the necessity of RAB6 for cell maintenance, we generated 3D organoids from *Rab6a* KO mice that had not undergone induction. These organoids were subsequently subjected to RAB6 deletion through 4-OHT treatment *in vitro*, and their development was monitored for 2 days post-passaging. Remarkably, after just one round of subculturing, RAB6-depleted organoids exhibited a significant reduction in the formation of new crypts, and most of them died (Fig. 5B; Movies 1 and 2). Collectively, these findings underscore the vital role of RAB6 in the self-renewal and maintenance of stem cells.

## DISCUSSION

Most of our knowledge of RAB function in mammals comes from studies performed with cultured cells. However, the available mouse models have been instrumental in clarifying the function of several RAB proteins, including RAB3, RAB6, RAB8, RAB10 and RAB11, in a physiological context (Homma et al., 2021). A previous study investigated the function of RAB6 in intestinal epithelial cells using conditional deletion of the *Rab6a* gene (Iwaki et al., 2020). In contrast to our results, that study reported no embryonic lethality; however, the pups died shortly after birth as a result of massive lipid accumulation. This discrepancy between the two studies could be due to differences in the genetic background of the mice used. Indeed, the genetic background can impact the mutant phenotypes, as described, e.g. in the case of targeted disruption of the mouse EGF receptor (Threadgill et al., 1995). In this study, we used mice backcrossed into the B6N background for more than ten generations. Unfortunately, the mouse background was not mentioned in the Iwaki study (Iwaki et al., 2020).

In our study, we observed two major effects of RAB6 depletion: impaired migration of intestinal epithelial cells along the villi and reduced stem cell number. Interestingly, these defects occurred without significant disruption of the epithelial architecture or the apico-basal polarity of cells. This aligns with reported similar observations following the depletion of RAB6 in mammary luminal cells, where the localization of both apical and basolateral markers remained unaffected in *Rab6a* KO cells (Cayre et al., 2020). The role of RAB6 in trafficking pathways in polarized cells has yet to be fully elucidated. In *Drosophila* photoreceptors, RAB6 is required for apical but not basolateral transport (Iwanami et al., 2016; Satoh et al., 2016). Our results suggest that, in mouse intestinal epithelial cells, RAB6 may not be essential for apical transport, or that the loss of RAB6 could be compensated for by another exocytic RAB, for instance, RAB8.

The migratory defect we observed in this study could result from several causes. Given that RAB6 is involved in recycling inactive β1 integrin through the Golgi complex (Shafaq-Zadah et al., 2016), we tested such a role in RAB6-depleted organoids. Although we found no clear evidence that RAB6 depletion impairs the recycling of internalized β1 integrin, we did notice depletion of β1 integrin from the basolateral membrane in *Rab6a* KO cells, and a decrease in total protein level. These data suggest that loss of RAB6 might impact the transport of newly synthesized β1 integrin to the basolateral membrane, leading to its degradation in lysosomes. An alternative explanation for the observed migratory defect could involve changes in the basement membrane composition in *Rab6a* KO mice. The intestinal epithelial basement membrane exhibits variability in laminin composition, with a laminin-5 gradient from the top to the bottom of the villus (Felsenthal and Vignjevic, 2022; Glentis et al., 2014), potentially serving as a cue for the directional migration of cells. It was recently shown that the secretion of laminin and other extracellular matrix components is severely impaired in RAB6-depleted MDCK cells (Homma et al., 2019), consistent with its role in the post-Golgi secretory pathway (Fourriere et al., 2019). In support of this hypothesis, we observed that *Rab6a* KO cells exhibited weaker attachment to the basement membrane compared with control mice. However, collagen IV or laminin α5 (part of laminin 10) were not affected in *Rab6a* KO gut epithelium.

An unexpected finding was that RAB6 depletion decreased the number or function of intestinal stem cells. However, at the same time, there was an increase in cell proliferation in the crypts, implying that RAB6 loss does not affect transit-amplifying cells. The loss of RAB6 also did not affect cell differentiation, as there were no effects on Paneth and goblet cell number or their phenotype. Thus, RAB6 loss did not impair their secretory process. The underlying importance of RAB6 in stem cell function remains to be fully elucidated. A plausible hypothesis is that RAB6 loss disrupts the Notch signaling pathway, which plays a crucial role in the maintenance of stem cells (Fre et al., 2005; van Es et al., 2005). Interestingly, in a genetic screen, warthog, the *Drosophila* homolog of RAB6 (DRAB6), was identified as a Notch modifier (Purcell and Artavanis-Tsakonas, 1999), suggesting a potential mechanism whereby the loss of stem cells in our system could result from a defect in Notch transport, thereby affecting Notch signaling. In addition, a recent study identified a miR-252-5p-RAB6 regulatory axis, which is involved in *Drosophila* wing development by controlling the Notch signaling pathway (Lim et al., 2023).

In conclusion, our findings emphasize the indispensable role of RAB6 in the development of the gut epithelium, as well as its renewal in homeostasis, suggesting its potential significance in gastrointestinal pathologies.

## MATERIALS AND METHODS

### Mouse models

*Rab6a*^loxP/loxP^ mutant mice (Bardin et al., 2015) were crossed with *Rab6b*^−/−^ mice to generate *Rab6a*^loxP/loxP^; *Rab6b*^−/−^. Animals of both sexes were used. Then, these animals were crossed with transgenic mice expressing Cre recombinase under the control of the villin or the Villin-CreER^T2^ promoter (el Marjou et al., 2004). The *Itgb1*^loxP/loxP^ strain was crossed with transgenic mice expressing Cre recombinase under the control of the Villin-CreER^T2^ promoter (el Marjou et al., 2004).

To induce Cre recombinase activity, mice were injected intraperitoneally with tamoxifen (20 mg/kg, Sigma-Aldrich, T5648) for three consecutive days for the *Rab6a* Villin-CreER^T2^ model, or five consecutive days (at 40 mg/kg) for the *Itgb1* Villin-CreER^T2^ model.

All experiments using mice were carried out according to the recommendations of the European Community (2010/63/UE). The animals were bred and cared for in the Specific Pathogen Free Animal Facility of Institut Curie (agreement E75-05-18). All animal procedures were approved by the ethics committee of the Institut Curie CEEA–IC #118 and by the French Ministry of Research (26880-20200813165686).

### Antibodies

For immunofluorescence experiments, the following antibodies were used: mouse anti-CD29 (1:100, BD Biosciences, 610467), goat anti-DPPIV (1:100, R&D Systems, AF1180), goat anti-collagen type IV (1:2000, Sigma-Aldrich, AB769), rat anti-ZO-1 (1:100, Sigma-Aldrich, MABT339), rat anti-E-cadherin (1:100, Invitrogen, 13-1900), rabbit anti-LAMA5 (1:500, Sigma-Aldrich, SAB4501720), rabbit anti-Ezrin (1:100; Zwaenepoel et al, 2012), rabbit anti-lysozyme (1:1000, Dako, A0099), rabbit anti-cleaved caspase 3 (1:100, Cell Signaling Technology, 9661), rabbit anti-OLFM4 (1:200, Cell Signaling Technology, D6Y5A), rabbit anti-Mucin 2 (1:200, Santa Cruz Biotechnology, sc-15334), rabbit anti-phospho-histone H3 (1:500, Sigma-Aldrich, 06-570), rabbit anti-Rab6 (1:100, Santa Cruz Biotechnology, sc-310), Alexa Fluor 488 rabbit anti-Na^+^-K^+^ATPase (1:200, Abcam, ab197713), Alexa Fluor 488 anti-Phalloidin (1:200, Invitrogen, A12379). All Alexa Fluor 488- or 647-conjugated secondary antibodies were from Jackson ImmunoResearch Laboratories and used at 1:200 (711-545-152, 711-605-152, 715-545-150, 715-605-150, 712-545-153, 712-605-153, 705-605-147, 705-545-147).

For western blotting, the following antibodies were used: mouse anti-CD29 (1:500, BD Biosciences, 610467), homemade affinity-purified rabbit anti-RAB6 (1:1000; Goud et al., 1990), rabbit anti-tubulin from the Recombinant Antibody Platform of the Institut Curie (1:500; https://science.institut-curie.org/platforms/therapeutic-recombinant-antibodies), rabbit anti-actin (1:500, Sigma-Aldrich, A4700). All secondary antibodies coupled to horseradish peroxidase were from Jackson ImmunoResearch and used at 1:10,000 (711-035-152, 715-035-150).

### Experiments using small intestine tissue

#### Whole-mount immunofluorescence staining

The small intestine (jejunum part) was isolated, flushed gently with cold 1× PBS to remove fecal content, hand cut into 1 cm pieces and fixed with 4% paraformaldehyde (PFA) in 1× PBS for 1 h at room temperature (RT). The samples were cut with vibratome to obtain 300-μm-thick gut slices. For immunostaining, slices were permeabilized with 1% Triton X-100 (Sigma-Aldrich) in PBS for 1 h at RT, blocked with 0.2% Triton X-100, 1% bovine serum albumin, 3% horse serum in PBS for 1 h at RT. Slices were then incubated with primary antibodies overnight at 4°C. Sections were washed three times 1 h with 0.2% Triton X-100 in PBS, with mild shaking at RT and then incubated with Alexa-conjugated secondary antibodies and DAPI (1:10,000, Sigma-Aldrich) overnight at 4°C. Sections were washed three times for 1 h each with 0.2% Triton X-100 in PBS at RT. Sections were mounted on SuperFrost Plus Adhesion slides (Epredia, J1800AMNZ) with Aqua-Poly/Mount (Polyscience) overnight at RT.

#### Histology

Small intestine tissues were fixed in 4% PFA overnight at 4°C, then incubated in 30% sucrose solution for 1 day at 4°C and embedded in Tissue-Tek OCT (Sakura Finetek) before freezing. Frozen sections of 15 μm thickness were obtained using a Leica CM1950 cryostat and processed for Hematoxylin/Eosin staining. Images were acquired using 10× dry objective on Nikon ECLIPSE Ti2 inverted microscope and Nikon imaging software.

#### Migration and proliferation assays

For migration and proliferation experiments, EdU staining was performed using the Click-iT EdU kit (Invitrogen, C10340), according to the manufacturer's recommendations. To assess *in vivo* migration, mice were injected with EdU (30 μg/g mouse; Sigma-Aldrich, 900584) 72 h before sacrifice. For *in vivo* proliferation experiments, mice were injected with EdU 2 h before sacrifice.

For the migration assay, quantification of the distance traveled by enterocytes was performed as follows. The orientation and crypt position of each villus were manually identified by hand and reoriented to a perpendicular position with Fiji's rotate tool. The EdU fluorescent channel was segmented using the ‘MakeBinary’ macro for Fiji. The vertical position of all foreground pixels of the segmented image, corresponding to regions of EdU fluorescence, was collated into a violin plot, representing the distribution of cell travel distance.

#### Detachment assay and immunoblotting

The small intestine (ileum part) was isolated, cleaned with cold 1× PBS and opened longitudinally. The intestine was placed in ice-cold dissociation reagent 1 (PBS, 30 mM EDTA, 1.5 mM DTT) at 4°C for 20 min. Then, the tissue was transferred to prewarmed dissociation reagent 2 (PBS, 30 mM EDTA) and incubated at 37°C with mild rocking for 12 min. Then, epithelial cells were dissociated by shaking the tube vigorously 20 times. Cells were pelleted by centrifugation at 1200 ***g*** for 5 min at 4°C and then immediately frozen with liquid nitrogen for storage. For ITGB1 samples, pellets were lysed in a buffer containing 50 mM Tris-HCl, 150 mM NaCl, 0.5% Triton X-100, 1% sodium dodecyl sulfate (SDS; Euromedex), 5% glycerol, and protease inhibitor cocktail (PIC, Sigma-Aldrich) and then sonicated five times for 10 s each time at 4°C. For RAB6 samples, pellets were lysed in RIPA buffer [50 mM Tris-HCl pH 7.4, 50 mM NaCl, 5 mM EDTA, 0.5% Nonidet P-40 (NP-40, Euromedex), 1× PIC] and cells were passed eight times through a 25 G needle, then kept under agitation for 2 h at 4°C. All the samples were centrifuged at 14,000 rpm (18,000 ***g***) for 15 min at 4°C and supernatants were collected. Protein concentrations were determined using the Bradford assay.

For the detachment assay, protein concentration was compiled in a graph. For immunoblotting, samples were mixed with Laemmli buffer and boiled for 5 min at 95°C. Then 100 μg (for ITGB1 samples) or 50 μg (for RAB6 samples) of proteins were loaded on 7.5% or 12% SDS polyacrylamide gels and transferred onto nitrocellulose membranes (Bio-Rad). Membranes were blocked with 5% non-fat dried milk, 0.1% Tween detergent in PBS for 30 min at RT. Membranes were then incubated with primary antibodies overnight at 4°C, followed by incubation with horseradish peroxidase-conjugated secondary antibodies for 1 h at RT. Detection was performed by using SuperSignal West Pico or Femto chemiluminescent substrate (Thermo Fisher Scientific) and ChemiDoc imaging system with Image Lab software (Bio-Rad).

### Crypt isolation, organoid culture and RAB6 depletion *in vitro*

Intestinal crypts were isolated as previously described (Baghdadi and Kim, 2022). At the end of the procedure, isolated crypts were mixed with Matrigel (Corning) and ENR media [DMEM/F-12 GlutaMAX (Gibco), 1% penicillin and streptomycin (Gibco), 10 mM HEPES (Sigma-Aldrich), 1× B-27 (Gibco), 1× N-2 (Gibco), 50 ng/ml of human EGF (Sigma-Aldrich), 100 ng/ml noggin (R&D Systems), 500 ng/ml mouse R-spondin (R&D Systems)] at 1:1 ratio. Then, 50 μl drops were plated on a 24 well-plate.

To deplete RAB6 *in vitro*: 2 days after crypt isolation, organoids were treated once with 2 μM of 4-OHT (Sigma-Aldrich, H7904), diluted in methanol or with methanol/PBS (used as control).

To follow the development of secondary organoids (Fig. 5B), 5 days after crypt isolation subculturing of organoids was performed by passing organoids through a 20 G needle until a complete dissociation. Crypts were then plated again on a 24 well-plate and imaged by live imaging using a ZEISS Celldiscoverer 7 imaging system (20× dry objective); images were acquired every hour for 30 h.

To follow the development of primary organoids (Fig. 5A), organoids were generated from mice treated or not with tamoxifen for 3 days. Intestinal crypts were imaged using an EVOS M5000 microscope (4× dry objective) at t=0 h and t=15 h after plating.

### Experiments using organoids

Isolated crypts were plated on 24-well-plates as described above and treated or not once with 2 μM of 4-OHT.

#### 3D organoid immunofluorescence staining

Seventy-two hours after RAB6 depletion, organoids were fixed with 4% PFA for 20 min at RT. Permeabilization was carried out with 0.2% Triton X-100 in PBS for 10 min at RT, washed three times in PBS for 5 min, and blocked for 20 min with 0.2% Triton X-100, 1% fetal bovine serum in PBS. Incubations with primary and secondary antibodies were performed with 0.2% Triton X-100 in PBS overnight at 4°C. Organoids were mounted on the slide using Aqua-Poly/Mount.

#### 3D organoid immunoblotting

Media was removed and replaced by fresh, cold 1× PBS for 5 min. Organoids were collected by pipetting up and down and centrifuged at 150 ***g*** for 10 min at 4°C. Pellets were resuspended in RIPA buffer (50 mM Tris-HCl pH 7.4, 150 mM NaCl, 1% NP-40, 0.1% SDS, 0.5% deoxycholate), vortexed for 30 s and centrifuged at 150 ***g*** for 1 min at 4°C. Supernatants were collected and 20 μg of proteins were loaded on the gel and processed for western blotting.

#### Integrin recycling in organoids

Organoids were collected in fresh, cold 1× PBS and centrifuged at 300 ***g*** for 5 min at 4°C. Pellets were then resuspended with anti-CD29 antibody diluted in ENR media and incubated on ice for 30 min to allow surface staining of β1 integrin. Then, pre-warmed ENR media was added and tubes were placed at 37°C for 20 min to allow endocytosis of labeled integrins. To remove surface-bound antibodies, acid wash (0.2 M HCl-glycine pH 2.2) was performed for 2 min at 4°C and directly neutralized by adding 1 M Tris. Then, pre-warmed ENR media was added, and tubes were placed at 37°C for 30 min to allow the recycling of β1 integrin back to the surface. At each step, samples were finally fixed with 4% PFA, permeabilized, incubated with secondary antibodies+DAPI and mounted on a slide.

### Confocal imaging

Confocal images displayed in Figs 1, 2, 3, 4 and Figs S1, S2, S3, S4 and S5 were acquired on Zeiss LSM880 NLO inverted laser scanning confocal equipped with Airyscan module, using a 63×/1.4, 40×/1.3 and 25×/0.8 oil objectives. Airyscan *z*-stacks were processed using Zen software using the Airyscan processing routine.

### Image quantification

#### Villus and crypt length

The transition line between crypt and villus was drawn manually. Then, the length between this line and the top of the villus was measured using Fiji software to obtain the height of the villus, and the length of crypts was obtained by measuring the distance between this line and the bottom of crypt.

#### Lateral pool of β1 integrin in RAB6 tissues

β1 integrin and DAPI signals were determined using the Fiji thresholding command excluding regions <50-px to avoid non-specific signal. Regions of interest of 70 µm were defined and processed to line scans using Fiji software. Then, the measured mean fluorescence intensity of β1 integrin signal was normalized to the measured mean fluorescence intensity of the DAPI signal. Graphics show the mean fluorescence intensity from one section.

#### Recycling assay

Maximum intensity *z*-projections over five stacks were performed using Fiji. β1 integrin and DAPI signals were determined using the Fiji's thresholding command excluding regions <50-px to avoid non-specific signal. Regions of interest of 20 µm were defined and processed to line scans using Fiji software. Then, the measured mean fluorescence intensity of β1 integrin signal was normalized to the measured mean fluorescence intensity of DAPI signal. Quantification of the apical (zone 1), lateral (zone 2) and basal pool (zone 3) of β1 integrin following the recycling assay in *Rab6a* Ctrl or KO gut organoids was performed as explained in Fig. S4B. Graphics show the mean fluorescence intensity over five sections.

### Statistical analysis

All statistical analyses were performed using GraphPad Prism 9.5 software. Each dot on the graph corresponds to a mouse. *P*-values were determined using two-tailed unpaired Student's *t*-test or Mann–Whitney test. Error bars correspond to s.d. Detailed numbers of villi, crypts and mice used for statistical analysis are given in Table S2.

## Supplementary Material

10.1242/develop.203038_sup1 Supplementary information
