# Supplementary material for: The GTPase RAB6 is required for stem cell maintenance and cell migration in the gut epithelium
Source: Development. 2024 Oct 21;151(21):dev203038. doi: 10.1242/dev.203038 (PMC11529276; doi:10.1242/dev.203038)
Supplement: Supplementary information [file develop-151-203038-s1.pdf]

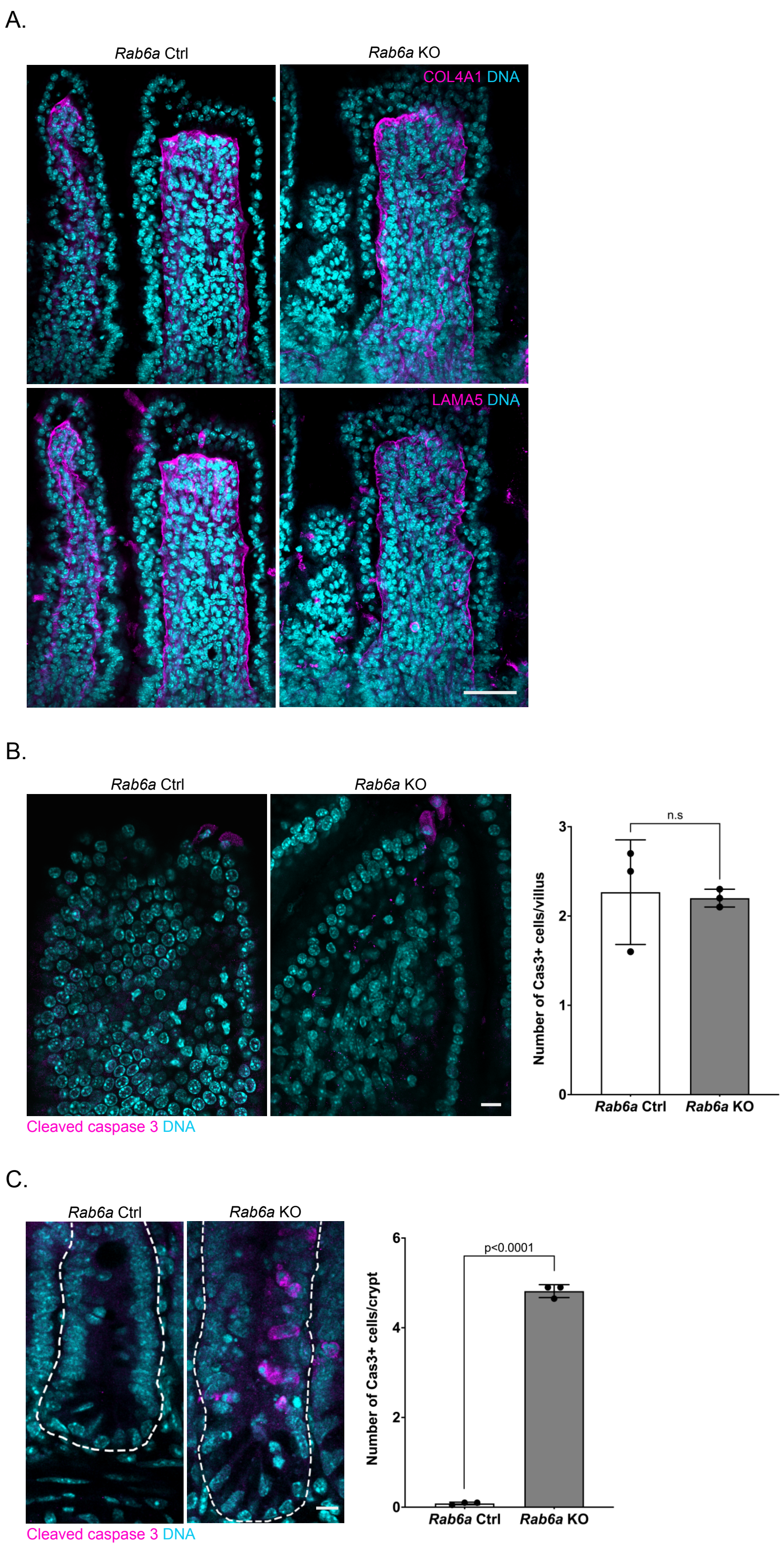


**Fig. S1. RAB6 depletion does not affect basement membrane organization. RAB6 depletion increases apoptosis in crypts but not in villi**

(**A**) Immunostaining of collagen Type IV (COL4A1) and laminin 10 (LAMA5) in Control and *Rab6a* KO mice. Scale bar: 50µm. (**B, C**) Immunostaining and quantification of cleaved caspase 3 (Caspase 3 +) in villi (**B**) and crypts (**C**) in *Rab6a* Ctrl and *Rab6a* KO mice (n=30 villi and 60 crypts). Scale bar: 10µm. Data were analyzed by Student's t-test; error bars indicate standard deviation (SD).

Development • Supplementary information

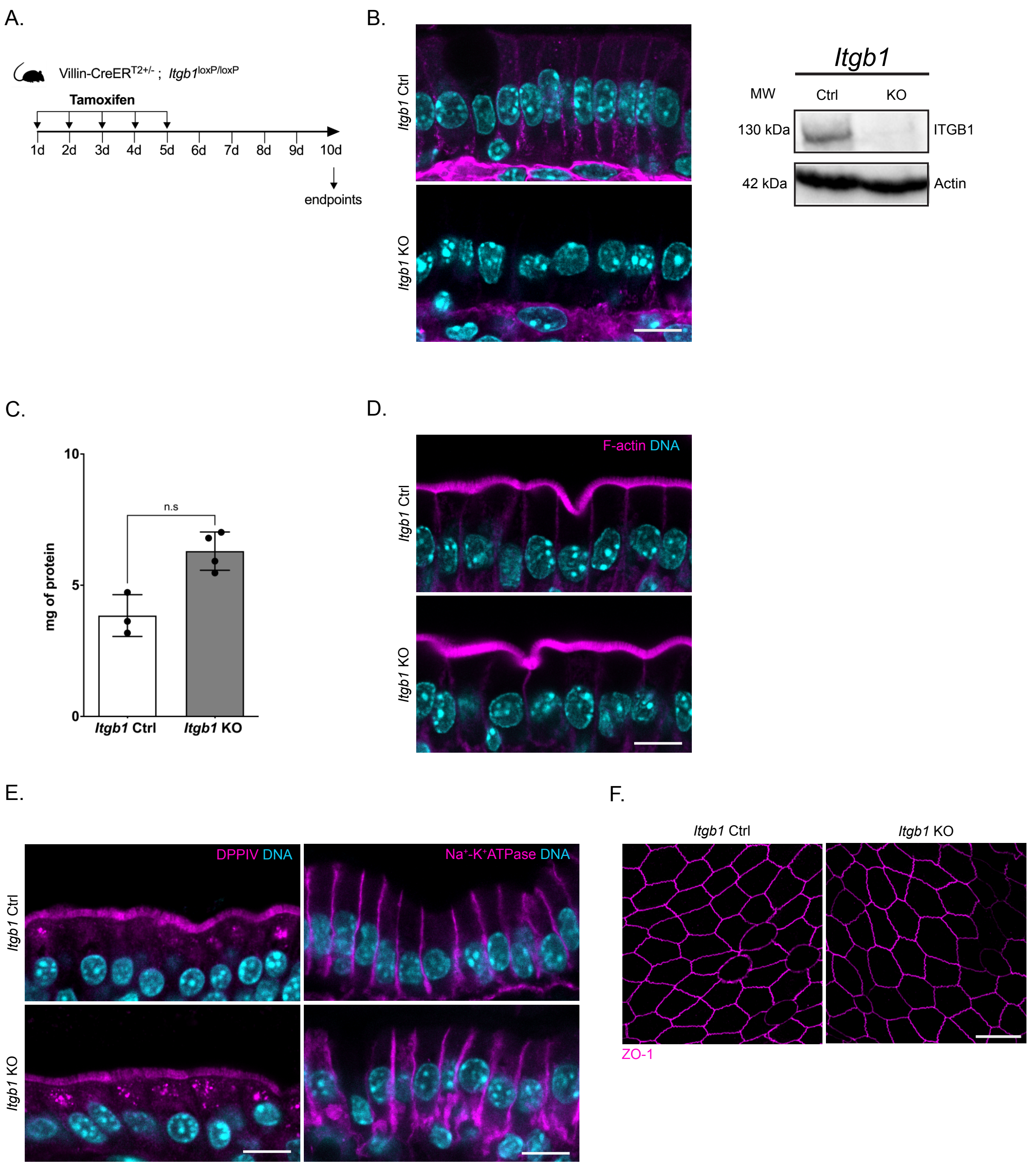


**Fig. S2. ß1 integrin depletion in the gut displays effects close to that observed in Rab6a KO mice**

(**A**) Experimental scheme for ß1 depletion. Mice were injected intraperitoneally with Tamoxifen once per day for 5 consecutive days and sacrificed at day 10 (**B**). Validation of ß1 integrin depletion by immunostaining (left) and western blotting (right); actin was used as a loading control; MW= Molecular weight; Scale bar: 10µm. **C**) In order to assess cell adhesion strength, protein concentration of epithelial cell lysates from small intestines tissues was quantified using the detachment assay. Data were analyzed by Mann-Whitney test; error bars indicate standard deviation (SD); (**D**) Epithelium in control and Rab6 KO mice, stained for F-actin (magenta) and DNA (cyan), Scale bar: 10µm. (**E**) Immunostaining of DPPIV (apical) and Na$^+$-K$^+$ATPase (lateral) polarity markers. Scale bar: 10µm; (**F**) Immunostaining of tight junction marker ZO-1. Scale bar: 10µm.

Development • Supplementary information

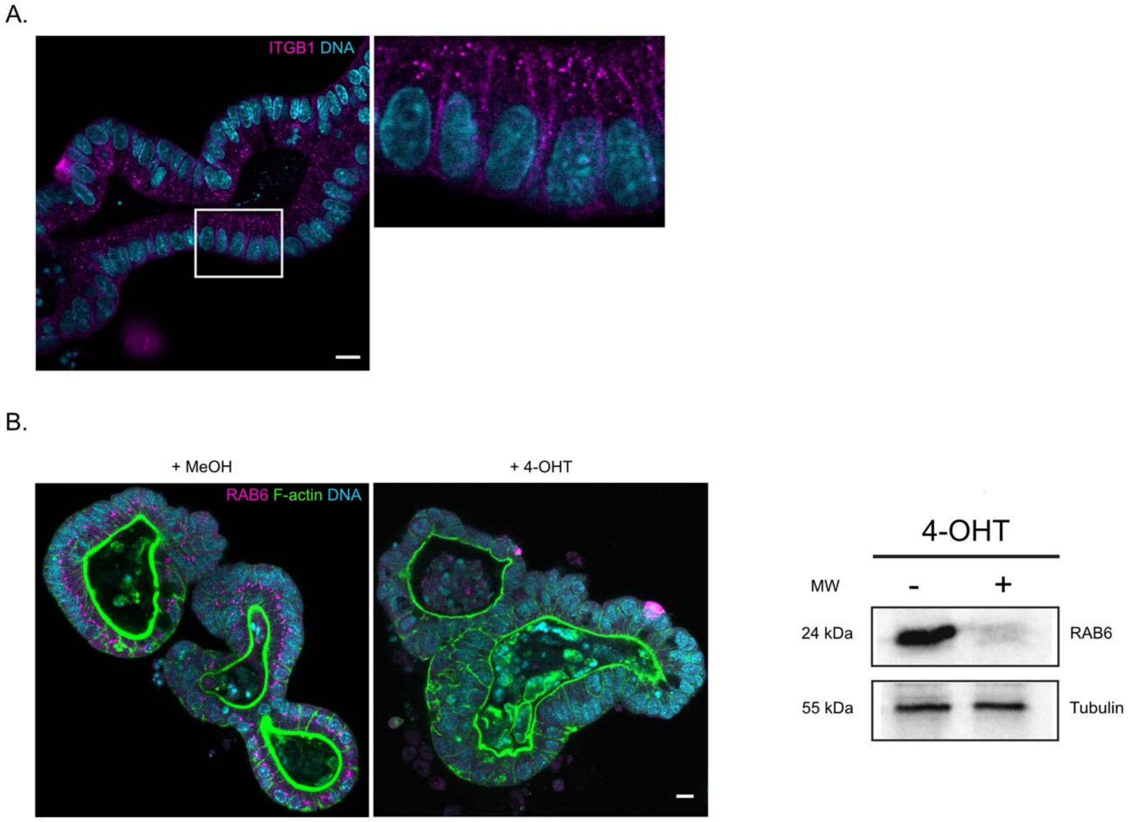


**Fig. S3. Depletion of RAB6 in gut organoids and localization of ß1 integrin**

(**A**) Immunostaining of ß1 integrin in *Rab6a* KO gut organoids shows basolateral and intracellular pool of ß1 integrin. Scale bar: 10µm; (**B**) RAB6 depletion *in vitro* in gut organoids by immunostaining and western-blotting; tubulin was used as a loading control; MW= Molecular weight. Scale bar: 10µm.

Development • Supplementary information

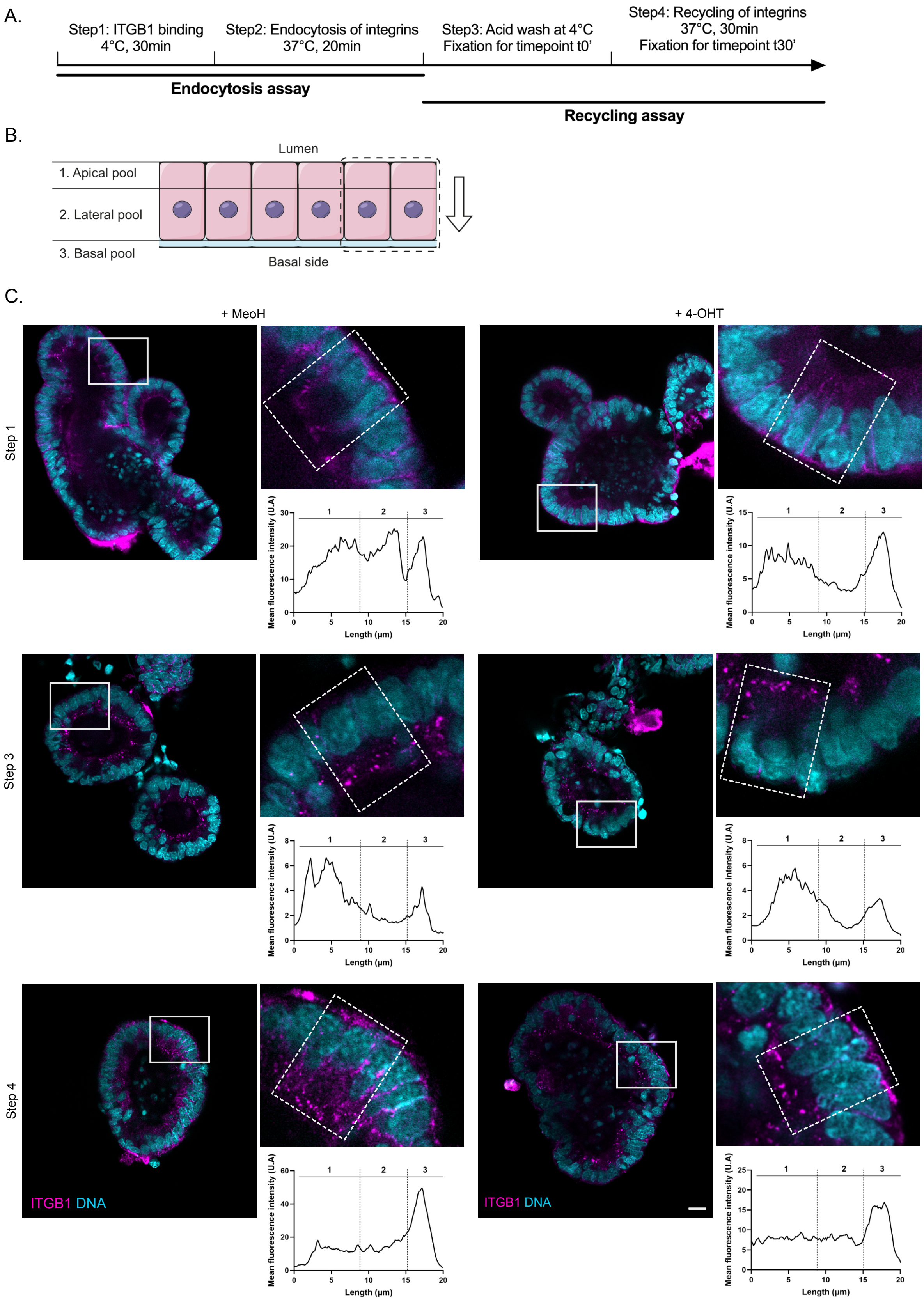


**Fig. S4. Integrin recycling assay**

(**A**) Experimental scheme for integrin recycling assay. (**B**) For quantification, the distribution of β1 integrin was divided in 3 zones: apical (zone 1), lateral (zone 2) and basal pool (zone 3). (**C**) Representative images of steps 1, 3 and 4 of the integrin recycling assay in *Rab6a* KO and control organoids stained for ß1 integrin. Corresponding quantifications of regions of interest (dashed areas) are displayed for each step of the recycling assay. Scale bar: 10µm

Development • Supplementary information

A.

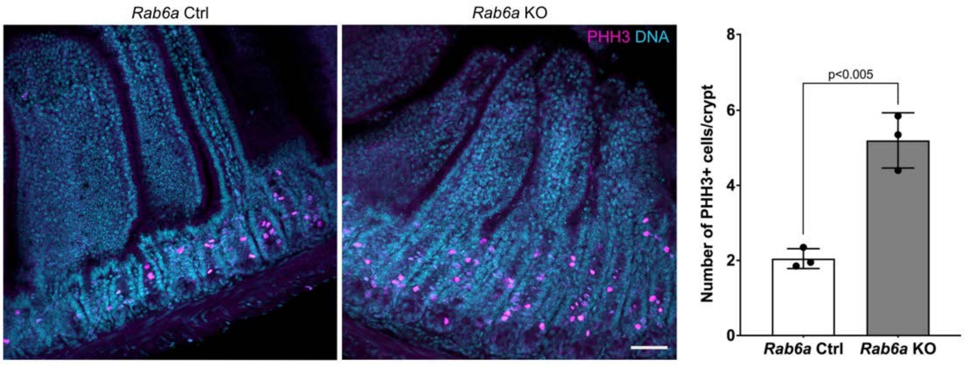


**Fig. S5. Rab6 depletion leads to increased number of proliferative cells**

Immunostaining and quantification of Phospho-histone 3 (PHH3+) in *Rab6a* Ctrl and *Rab6a* KO mice (n=60 crypts). Scale bar: 50µm. Data were analyzed by Student's t-test; error bars indicate standard deviation (SD).

## Table S1. RAB6 depletion in the gut is embryonic lethal

| | *Rab6A*$^{+/+}$ | *Rab6A*$^{+/-}$ | *Rab6A*$^{-/-}$ | Total |
|---|---|---|---|---|
| Obtained | 36 | 24 | 0 | 60 |
| Obtained (%) | 60 | 40 | 0 | 100 |
| Expected (%) | 50 | 25 | 25 | 100 |

The table shows the genotype distribution of mice born from a cross between a *Rab6a*$^{loxP/loxP}$ male and two *Rab6a*$^{loxP/WT}$ Villin-Cre$^{+/-}$ females. Data were analyzed by binomial test, *P*-value=4.162e−08.

Development • Supplementary information

**Table S2. Overview of the number of villi, crypts and mice used for quantifications**

| Figure | Numer of villi or crypts counted/mouse | Number of mice used | Total |
|---|---|---|---|
| 2C | n= 20 villi | n= 3 | n= 60 villi |
| 2E | | n= 4 Ctrl/ n=5 KO | |
| 3A | n= 10 villi | n= 3 | n= 30 villi |
| 3D | n= 10 villi | n= 3 | n= 30 villi |
| 4A | n= 25 crypts | n= 3 | n= 75 crypts |
| 4B | n= 20 crypts | n= 3 | n= 60 crypts |
| 4C | n= 20 crypts | n= 3 | n= 60 crypts |
| 4D | n= 20 crypts | n= 3 | n= 60 crypts |
| 4E | n= 10 villi | n= 3 | n= 30 villi |
| 5B | n= 10 crypts | n= 3 | n= 30 crypts |
| S1B | n= 10 villi | n= 3 | n= 30 villi |
| S1C | n= 20 crypts | n= 3 | n= 60 crypts |
| S2C | | n= 3 Ctrl/ n=4 KO | |
| S5A | n= 20 crypts | n= 3 | n= 60 crypts |

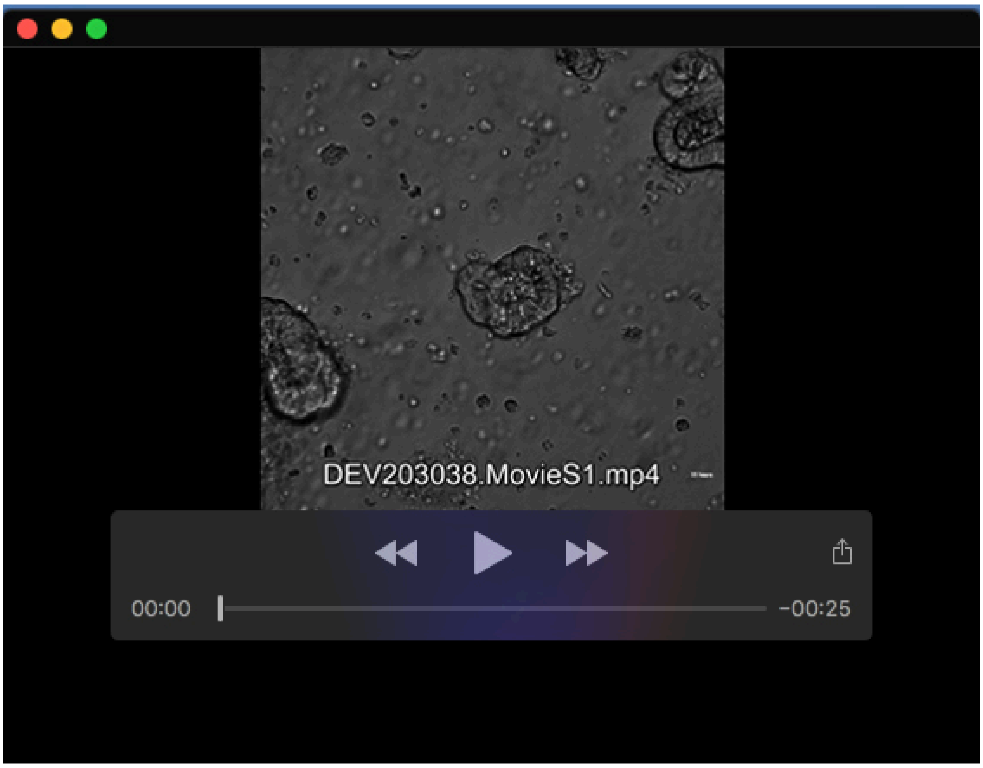


**Movie 1.** Movie showing the development of secondarsy organoids by live imaging from isolated crypts not depleted for RAB6.

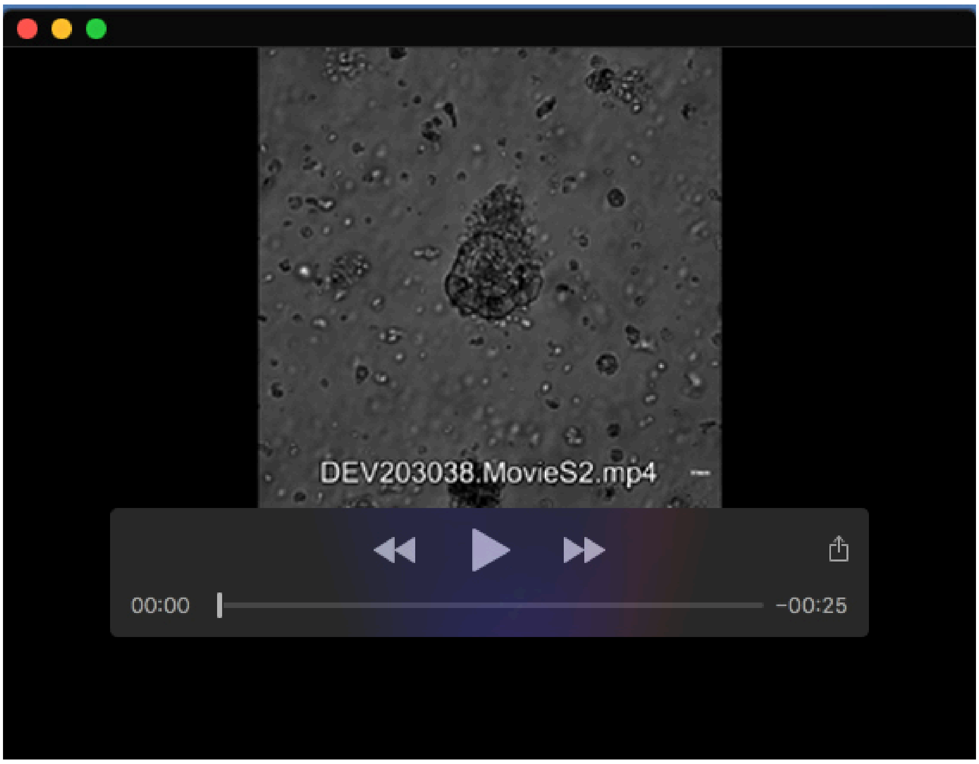


**Movie 2.** Movie showing the development of secondary organoids by live imaging from isolated crypts depleted *in vitro* for RAB6.
